# A Human-Like Senescence-Associated Secretory Phenotype Is Conserved in Mouse Cells Dependent on Physiological Oxygen

**DOI:** 10.1371/journal.pone.0009188

**Published:** 2010-02-12

**Authors:** Jean-Philippe Coppé, Christopher K. Patil, Francis Rodier, Ana Krtolica, Christian M. Beauséjour, Simona Parrinello, J. Graeme Hodgson, Koei Chin, Pierre-Yves Desprez, Judith Campisi

**Affiliations:** 1 Life Sciences Division, Lawrence Berkeley National Laboratory, Berkeley, California, United States of America; 2 Buck Institute for Age Research, Novato, California, United States of America; 3 Centre de Recherche du CHU Ste-Justine et Département de Pharmacologie, Université de Montréal, Montréal, Québec, Canada; 4 Department of Laboratory Medicine, Comprehensive Cancer Center, University of California San Francisco, San Francisco, California, United States of America; 5 California Pacific Medical Center Research Institute, San Francisco, California, United States of America; Roswell Park Cancer Institute, United States of America

## Abstract

Cellular senescence irreversibly arrests cell proliferation in response to oncogenic stimuli. Human cells develop a senescence-associated secretory phenotype (SASP), which increases the secretion of cytokines and other factors that alter the behavior of neighboring cells. We show here that “senescent” mouse fibroblasts, which arrested growth after repeated passage under standard culture conditions (20% oxygen), do not express a human-like SASP, and differ from similarly cultured human cells in other respects. However, when cultured in physiological (3%) oxygen and induced to senesce by radiation, mouse cells more closely resemble human cells, including expression of a robust SASP. We describe two new aspects of the human and mouse SASPs. First, cells from both species upregulated the expression and secretion of several matrix metalloproteinases, which comprise a conserved genomic cluster. Second, for both species, the ability to promote the growth of premalignant epithelial cells was due primarily to the conserved SASP factor CXCL-1/KC/GRO-α. Further, mouse fibroblasts made senescent in 3%, but not 20%, oxygen promoted epithelial tumorigenesis in mouse xenographs. Our findings underscore critical mouse-human differences in oxygen sensitivity, identify conditions to use mouse cells to model human cellular senescence, and reveal novel conserved features of the SASP.

## Introduction

Cellular senescence was first identified as a process that limits the proliferation (growth) of human cells in culture [1]. These early experiments showed that cultured human fibroblasts gradually lose proliferative capacity (arrest growth) until all cells in the culture cease division. Much of this growth arrest is now known to occur because most human cells do not express telomerase. Consequently, with each cell cycle, telomeres shorten and eventually fail, generating a persistent DNA damage signal that permanently arrests growth [2]. Subsequent studies showed that non-telomeric DNA damage, and many other stressors, can also induce senescence [3]. Indeed, cells from laboratory mice, which have long telomeres and often express telomerase, also show only limited growth in culture, but arrest because they accumulate oxidative DNA damage under standard culture conditions [4].

Cellular senescence is now recognized as a crucial tumor suppressor mechanism and formidable barrier to malignant progression [3], [5], [6]. The hallmark of senescent cells is an essentially irreversible p53- and p16^INK4A^/pRb-dependent cell cycle arrest. Senescent cells differ from reversibly arrested quiescent cells in several ways. For example, senescent, but not quiescent, human fibroblasts fail to induce c-Fos in response to mitogen stimulation [7], and express a senescence-associated β-galactosidase (SA-βGal) [8]. Some senescent cells form distinctive heterochromatic foci (SAHFs) [9], [10], and many harbor markers of persistent DNA damage [2], [11], [12], [13], [14]. Senescent human cells also secrete many biologically active proteins, a phenotype we term the senescence-associated secretory phenotype (SASP) [15], [16].

The senescence response might be beneficial or deleterious, depending on the age of the organism [17]. To understand this apparent paradox, the SASP may be particularly important. Senescent cells increase with age in many mammalian tissues and are found at sites of age-related pathologies [8], [18], [19], [20], [21], [22]. The SASP includes inflammatory cytokines that are thought to drive aging and age-related disease [23]. Indeed, some SASP factors, when chronically present, can disrupt tissue structure and differentiation [24], and others can promote malignant phenotypes in nearby premalignant cells [15], [25], [26], [27]. On the other hand, some SASP factors may be beneficial. For example, some reinforce the senescence growth arrest in an autocrine manner [28], [29], [30], [31]. Others may allow damaged cells to communicate their compromised state [13] in order to stimulate tissue repair or limit pathology [32]. To date, only the SASPs of human cells have been well characterized [15], [27], [28], [30], [33], [34], [35], [36].

Mice are important models for understanding normal and pathological processes in humans, and mouse cells are widely used to study cellular senescence – in culture and *in vivo*. However, there are differences between human and mouse cellular responses [4], [37], [38]. In addition to different causes for limited growth in culture, mouse, but not human, cells readily acquire an unlimited division potential (immortalization) when certain genes are mutated. Moreover, mouse, but not human, cells often spontaneously immortalize in culture [39], [40], [41]. It is not known whether mouse and human cells differ in their SASPs, although senescent mouse cells were shown to secrete specific factors that can have systemic effects [29], [42].

The use of mice to model human disease requires understanding critical mouse-human differences, and defining conditions under which mouse cells accurately mimic human cells. We show here that the SASP is largely conserved between mouse and human fibroblasts, providing mouse cells are cultured in physiological oxygen; standard culture conditions of supraphysiological oxygen suppress the SASP. We further show that segments of the matrix metalloproteinase (MMP) gene cluster are SASP components in both species, and that CXCL-1 (KC/GRO-α) is a conserved SASP factor that promotes premalignant epithelial cell growth. Finally, we show that only mouse cells that develop a human-like SASP stimulate epithelial tumorigenesis *in vivo*. Our studies identify fundamental similarities and differences in senescent phenotypes between mouse and human cells, and describe new features of the SASP that contribute to its biological activities.

## Results

### Senescence-Associated Secretory Phenotypes (SASPs) of Mouse Fibroblasts

To identify proteins secreted by senescent mouse cells, we used primary fibroblasts from adult mammary glands (mBFs) or embryos (MEFs). We cultured mouse cells in either atmospheric (∼20%; standard culture condition) or physiological (3%) oxygen. In 20% oxygen, presenescent (PRE) cells underwent senescence (SEN) after 8–10 population doublings (PDs), as expected [4]. However, although ∼65% of these SEN cells expressed SA-βGal, ∼40% synthesized DNA, despite little or no increase in cell number (Table 1). Because this SEN arrest is driven by oxidative DNA damage [4], we term it SEN(OXI). Mouse cells proliferate for many more PDs in 3% oxygen, as expected of cells with telomerase and long telomeres [4]. Nonetheless, they undergo an efficient senescence arrest in response to high dose (10 Gy) ionizing (X-ray) radiation (XRA). SEN(XRA) mouse cells also expressed SA-βGal (∼75%), but very few (<5%) synthesized DNA (Table 1). In this regard, SEN(XRA) mouse cells resembled SEN human cells, whether made senescent by replication in culture (REP) or XRA [15]. To compare PRE and SEN cells, we made PRE cells quiescent by culturing to confluence, thereby controlling for effects of cell proliferation (Table 1).

**Table 1:** 

Mouse cell strain	% O_2_	Culture condition	Growth status	Labeling Index	% SA-βGal	Designation
MEF (primary mouse embryonic fibroblasts)	3	presenescent, subconfluent	Growing	84	11	proliferating
	3	presenescent, cultured to confuency	Quiescent	16	nt	PRE
	3	X-ray-induced growth arrest	Senescent	4	74	SEN (XRA)
	20	presenescent, subconfluent	Growing	75	12	proliferating
	20	presenescent, cultured to confuency	Quiescent	15	nt	PRE
	20	growth arrest after repeated passage	Senescent	39	65	SEN (OXI)
mBF (primary adult mouse mammary gland fibroblasts)	3	presenescent, subconfluent	Growing	91	9	proliferating
	3	presenescent, cultured to confuency	Quiescent	15	nt	PRE
	3	X-ray-induced growth arrest	Senescent	4	76	SEN (XRA)
	20	presenescent, subconfluent	Growing	86	9	proliferating
	20	presenescent, cultured to confuency	Quiescent	13	nt	PRE
	20	growth arrest after repeated passage	Senescent	42	65	SEN (OXI)

**Figure 1:**
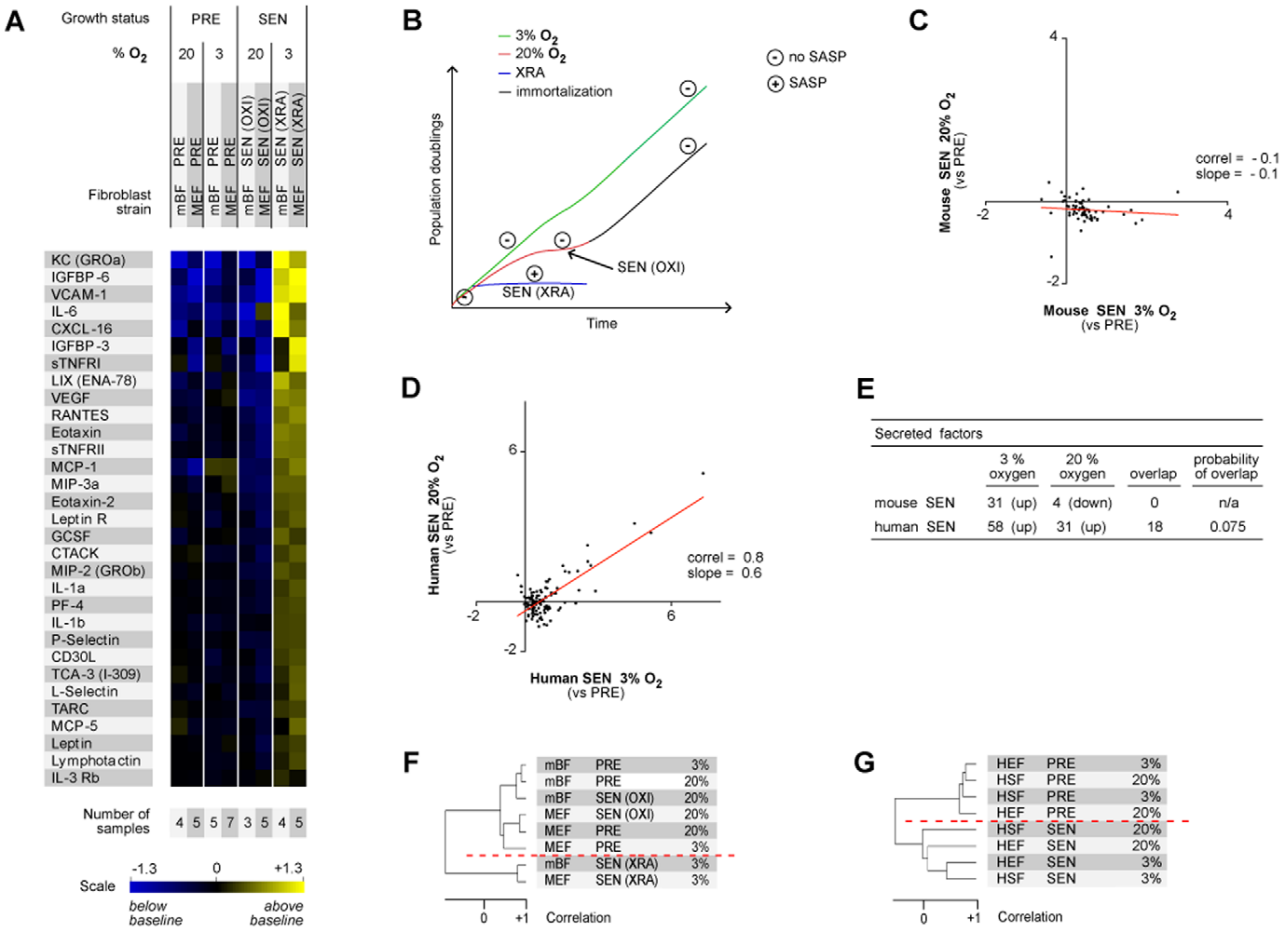
Secretory profiles of presenescent and senescent mouse fibroblasts. A) Soluble factors secreted by the indicted cells were detected by antibody arrays and analyzed as described [15]. For each cell strain, PRE and SEN signals were averaged and used as the baseline (average of PRE 3%, PRE 20%, SEN (XRA) 3%, SEN (OXI) 20%). Signals above baseline are shown in yellow; signals below baseline are in blue. The heat map key indicates log2-fold changes from baseline (changes greater than the scale show as saturated colors). The number of samples analyzed is shown below each lane (see also Dataset S1). B) Model of mouse cell proliferation in culture showing conditions under which SASPs do (+) and do not (−) develop. C–D) Correlation between the secretory profiles of mouse (C) or human (D) cells cultured and induced to senesce in 3% vs 20% O_2_. Baselines for the senescent profiles are the corresponding PRE profiles of cells cultured in the same O_2_ concentration. E) Comparisons of the number of secreted factors that change in human and mouse cells induced to senesce in 20% or 3% O_2_. F–G) Unsupervised hierarchical clustering analysis of PRE and SEN mouse (F) or human (G) fibroblasts (see also Fig. S1).

To determine the SASPs of murine cells, we collected serum-free conditioned media (CM) from PRE, SEN(OXI) and SEN(XRA) cells, normalized for cell number, and analyzed the CM using antibody arrays designed to detect 62 proteins selected for roles in intercellular signaling. We used a modified radioactivity-based detection protocol that greatly improved the assay's reliability and sensitivity [15]. We normalized each signal to control signals on every array to facilitate inter-sample and inter-experiment comparisons. For visual display (Fig. 1A; Fig. S1; Dataset S1), we expressed each secreted protein level as the log_2_ fold-change relative to a baseline derived from the average signal for that protein across all samples (all cell strains, growth conditions and oxygen concentrations).

MEFs and mBFs cultured in 3% O_2_ and induced to senesce by XRA secreted numerous proteins at significantly higher levels than similarly cultured PRE cells (Fig. 1A; Fig. S1A). In this regard, SEN(XRA) mouse cells resembled human cells made senescent by XRA and other means [15]. (Unless noted otherwise, we pooled data from four human fibroblast strains, two embryonic lung (HEF) and two neonatal foreskin (HSF) strains). Because the factors secreted by SEN(XRA) mouse cells overlapped substantially with the human SASP, we conclude that mouse cells express a human-like SASP, at least when induced to senesce at a physiological O_2_ concentration (Fig. 1B; see Fig. S1A–C for arrays, significance and comparisons).

In striking contrast, SEN(OXI) mouse cells secreted no factor at substantially higher levels than PRE cells, whether PRE cells were cultured in 20% or 3% O_2_ (Fig. 1A; Fig. S1A,B). Thus, SEN(OXI) cells, unlike SEN(XRA) cells in 3% O_2_, do not develop a SASP (Fig. 1B). In this regard, mouse cells differed markedly from human cells. That is, ambient O_2_ strongly influenced the secretory profile of SEN mouse cells, but had only minor effects on that of SEN human cells [15] (Fig. 1C–E). Further, the secretory profile of PRE mouse cells proliferating in 20% O_2_ bore little resemblance to that of PRE mouse cells proliferating in 3% O_2_ (Fig. S1D), whereas PRE human cells had similar profiles whether cultured in 3% or 20% O_2_ (Fig. S1D). In addition, PRE mouse cells proliferating in 20% O_2_ and then irradiated developed a weak SASP, compared to the robust SASP that developed in 3% O_2_ (Fig. S1A,B,E). SEN human cells, in contrast, developed robust SASPs, whether irradiated in 20% or 3% O_2_ (Fig. 1D; Fig. S1F).

Further analyses emphasized the strikingly different effects of O_2_ on proteins secreted by SEN mouse and human cells (Fig. 1E; Fig. S1). For mouse cells, SEN in 3% O_2_ [SEN(XRA)] resulted in significantly elevated secretion of 31 proteins (of 62 on the array). None of these 31 proteins (0 overlap) showed elevated secretion upon SEN in 20% O_2_ [SEN(OXI)], and 4 showed decreased secretion. For human cells, SEN in 3% O_2_ [SEN(REP or XRA)] resulted in significantly elevated secretion of 58 proteins (of 120 on the array). SEN in 20% O_2_ elevated 31 proteins, 18 of which overlapped with those that increased in 3% O_2_. Hierarchical clustering, which groups profiles on the basis of overall correlation [43], showed that, for mouse cells, the SEN(OXI) secretory profiles clustered with the PRE profiles, whereas the SEN(XRA) profiles formed an outlier cluster (Fig. 1F; Fig. S1B). By contrast, the profiles of human (HSF and HEF) fibroblasts clustered on the basis of their senescence status, rather than origin or the O_2_ concentration in which they were cultured (Fig. 1G). Thus, mouse cells that arrest growth in 20% O_2_ [SEN(OXI)] do not share secretory characteristics with SEN human cells, or mouse cells that senesce in physiological O_2_.

### Conservation between Human and Mouse SASPs

**Figure 2:**
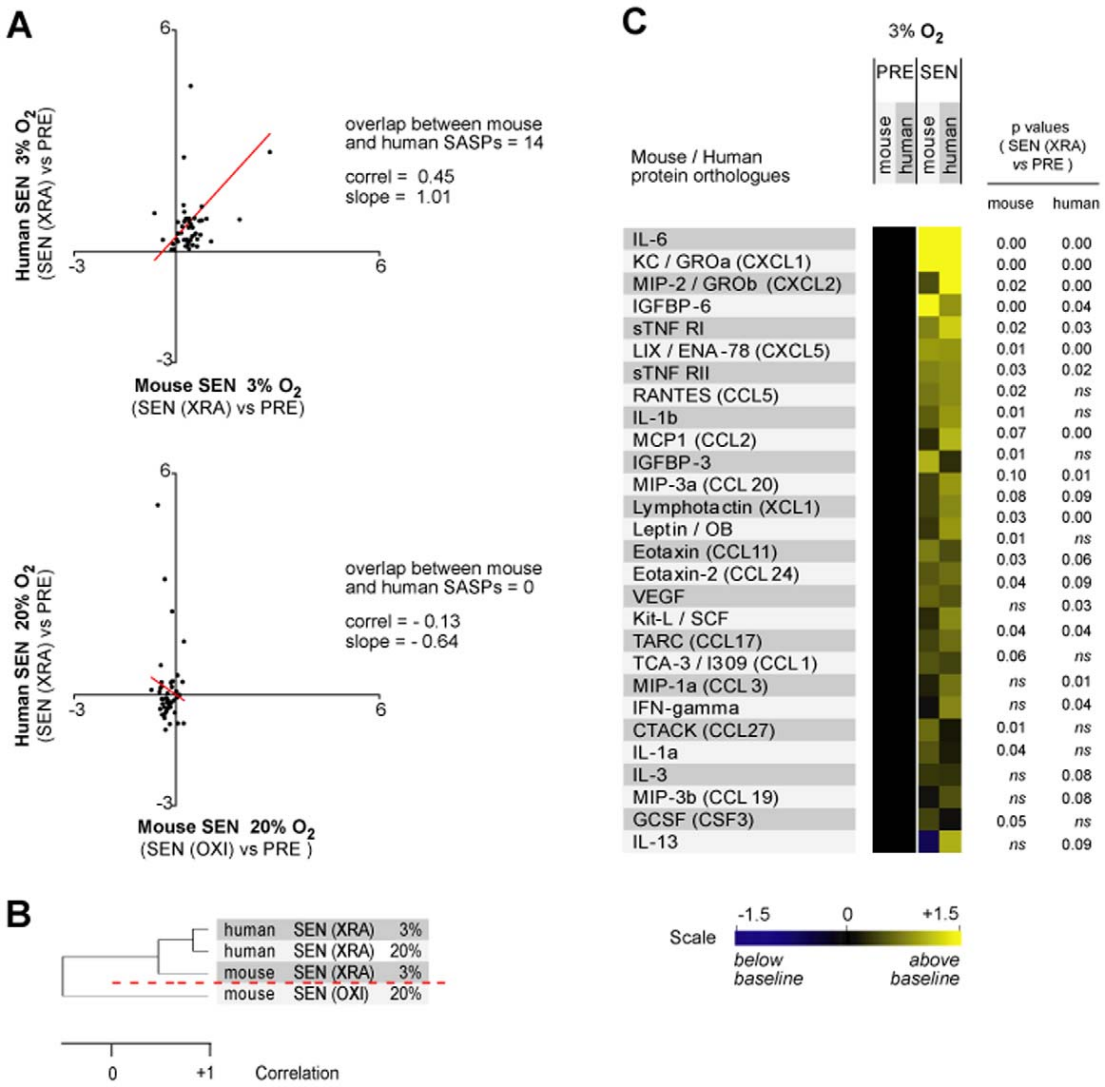
Orthology analyses of human and mouse secretory profiles. A) Correlation between human and mouse SEN cells cultured and induced to senesce in 3% O_2_ (top panel) and 20% O_2_ (lower panel). Baselines for each SEN profile are the secretory profiles of PRE cells cultured under the same O_2_ concentration (see Datasets S2, S3). B) Unsupervised hierarchical clustering analysis of cells in A. C) Direct comparisons of the secretory profiles of human and mouse cells cultured in 3% O_2_.

The human and mouse antibody arrays detect 42 orthologous proteins. Restricting analysis to these orthologs, we assessed the inter-species similarities and differences (Dataset S2, S3). The mouse SEN(XRA) (3% O_2_) profile was similar to the human SEN(XRA) (3% O_2_) profile when all orthologs were considered (Fig. 2A, upper panel). By contrast, the mouse SEN(OXI) (20% O_2_) profile bore little resemblance to the human SEN(XRA) or SEN(REP) (20% O_2_) profile (Fig. 2A, lower panel; Fig. S2A). Hierarchical clustering analysis again identified SEN(OXI) profiles as outliers (Fig. 2B). Ranking and probability of overlap analyses further supported the conclusion that only mouse cells cultured in 3% O_2_ develop a human-like SASP (Fig. S2B). Under these conditions, orthologs such as IL-6, CXCL-1 (GRO-α/KC) and IGFP-6 were similarly upregulated (Fig. 2C), whereas orthologs such as IL-2, IL-12 and CXCL-12 (SDF-1) were similarly unchanged (Fig. S2C). These analyses indicate that only mouse cells maintained under physiological O_2_ concentrations develop a SASP that is quantitatively and qualitatively similar to the human SASP.

### Effect of O_2_ on DNA Damage and Genomic Instability

**Figure 3:**
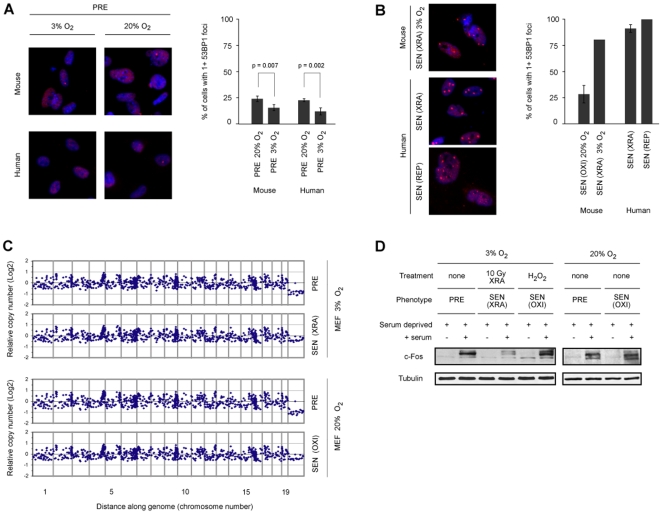
DNA damage, genomic instability and c-Fos response. A–B) DNA damage foci in mouse and human PRE and SEN fibroblasts cultured at 3% or 20% O_2_. Cells were immunostained for 53BP1 (red) and nuclei were stained with DAPI (blue). Bar graph shows the percentage of cells with 1 or more 53BP1 focus. C) Comparative genomic hybridization profiles of PRE and SEN mouse cells cultured at 3% or 20% O_2_. D) c-Fos response in mouse cells. PRE, SEN(XRA) or SEN(OXI) – induced either by incubation with 400 uM H_2_0_2_ or passage in 20% O_2_ – mouse cells were cultured at 3% or 20% O_2_, incubated in 0.5% serum for 48 h, then stimulated (+ serum) or not (−) with 10% serum. Cell lysates were prepared and analyzed by western blotting for c-Fos and tubulin (control) protein.

In human cells, the DNA damage response (DDR) drives the SASP and correlates with the presence of persistent DNA damage nuclear foci containing 53BP1 (p53 binding protein-1) [13]. To better understand O_2_-dependent differences between mouse and human cells, we assessed PRE and SEN cells for one or more (1+) 53BP1 foci (Fig. 3A,B; Fig. S3A). For both species, more PRE cells had 1+ 53BP1 foci when cultured in 20% O_2_ compared to 3% O_2_; the difference was small (5–10%) but significant. No PRE culture had >25% 53BP1-positive cells. When mouse cultures were induced to senesce in 3% O_2_ by XRA [SEN(XRA)], the fraction of cells with 1+ 53BP1 foci rose, similar to human cells induced to senesce by XRA or REP regardless of O_2_ concentration [13]. In contrast, mouse cultures induced to senesce by passage in 20% O_2_ [SEN(OXI)] showed no significant rise in cells with 1+ 53BP1. Further, the fraction of SEN(OXI) cells with 1+ 53BP1 foci was similar regardless of whether cells were synthesizing DNA (Fig. S3B; see Table 1). The outlier behavior of SEN(OXI) cells could not be explained by consistent chromosome losses or gains, at least at the resolution of 10–20 Mb, determined by comparative genomic hybridization (CGH). The CGH profiles of PRE and SEN mouse cells were identical, regardless of O_2_ concentration (Fig. 3C), as were the CGH profiles of PRE and SEN human cells (Fig. S3C). The results suggest that the SASP correlates with persistent DNA damage foci in mouse cells, as it does in human cells [13], and that the DNA damage caused by 20% O_2_ [4] does not generate the persistent DDR signaling needed for the SASP.

### Effects of O_2_ on the c-Fos Response

Quiescent human fibroblasts rapidly increase expression of the c-Fos proto-oncoprotein when stimulated by serum mitogens, but this response is lost upon senescence [4], [7]. Similarly, quiescent (PRE) mouse fibroblasts showed a robust c-Fos serum response, regardless of O_2_ concentration, whereas SEN(XRA) mouse cells (in 3% O_2_) did not (Fig. 3D). SEN(OXI) mouse cells, however, retained c-Fos inducibility (Fig. 3D). To determine how this retention related to the oxidative stress of 20% O_2_, we treated PRE mouse cells with H_2_O_2_, a strong oxidant. At the concentration used (400 µM), cells arrested growth and expressed SA-βGal [4], but retained c-Fos inducibility (Fig. 3D). Human cells cultured in either 3 or 20% O_2_ and treated with H_2_O_2_ behaved similarly [4]. Thus, mouse cells, like human cells, retain c-Fos inducibility when they arrest growth under severe oxidative stress, suggesting that SEN(OXI) is an analogous state, distinct from the senescent states of human cells or mouse cells in physiological O_2_.

### Conservation at mRNA and Intracellular Protein Levels

**Figure 4:**
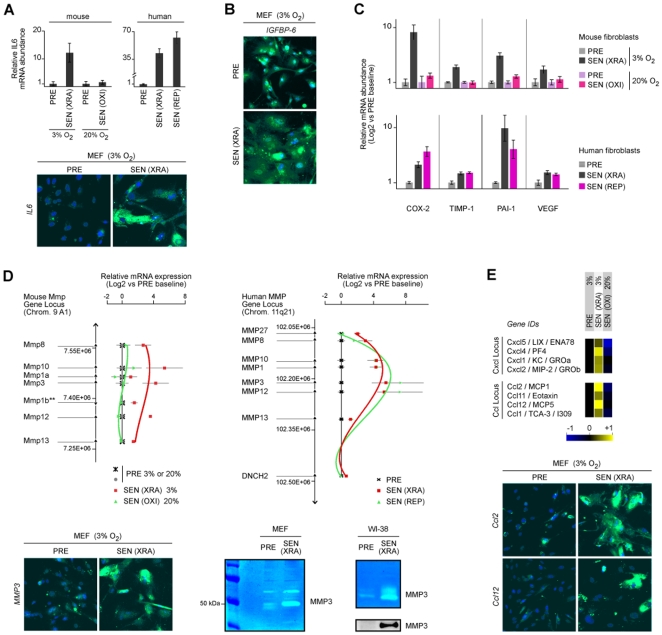
mRNA and intracellular protein expression and identification of MMP, CXCL and CCL loci as SASP components. A) IL-6 mRNA and intracellular protein in PRE and SEN mouse and human cells. IL-6 mRNA was quantified by RT-PCR (TaqMan). Intracellular protein was detected by immunofluorescence (green). Nuclei were stained with DAPI (blue). B) Intracellular IGFBP-6 protein was detected by immunofluorescence (green) of DAPI-stained (blue) cultures. C) mRNA levels of other senescence-associated genes (COX-2, TIMP-1, PAI-1 and VEGF) in mouse and human cells. mRNA was quantified by RT-PCR using TaqMan. D) Matrix metalloproteinases (MMP) are mouse and human SASP factors. Shown is the organization of the mouse (left) and human (right) MMP gene clusters. mRNA was isolated from mouse and human cells cultured as indicated: PRE cells in 3% (x) or 20% (gray dot) O_2_ (black line); SEN(XRA) cultured in 3% O_2_ (red line); SEN(OXI) mouse cells made senescent by passage in 20% O_2_ (green line); SEN(REP) human cells made senescent by passage in 20% O_2_ (green line). Abundance of the indicated MMP mRNAs was quantified by RT-PCR. PRE and SEN(XRA) mouse cells were immunostained for MMP-3 (bottom left). Conditioned media (CM) were assayed for MMP3 activity (zymography, bottom right) and protein level (western blotting, bottom right). E) Expression of Cxcl and Ccl gene clusters in PRE, SEN(XRA) and SEN(OXI) mouse cells. The genes are listed vertically in 5′ (top) →3′ order. Antibody arrays results are shown to the right. Bottom panels show immunostaining for intracellular Ccl2 and Ccl12.

Many human SASP proteins are upregulated at the level of mRNA abundance, and are detectable intracellularly by immunostaining [15]. We therefore determined mRNA and protein levels of the major SASP component IL-6 in PRE and SEN mouse cells. Compared with PRE cells, IL-6 mRNA (Fig. 4A, upper panel) and intracellular protein (Fig. 4A, lower panel) increased significantly when MEFs senesced in 3%, but not 20%, O_2_. Likewise, IGFBP-6, a prominent human SASP factor [15], increased intracellularly when MEFs senesced in 3% O_2_ (Fig. 4B), and mRNA levels of COX-2, TIMP-1, PAI-1 and VEGF increased upon senescence in human fibroblasts, and mouse fibroblasts in 3%, but not 20%, O_2_ (Fig. 4C).

### Members of MMP and Other Gene Clusters Comprise the Conserved SASP

To better understand the SASP and its conservation, we assayed CM for matrix metalloproteinases (MMPs), which were not assayed by the cytokine antibody arrays. MMP genes are of particular interest because several are clustered in syntenic regions on mouse chromosome 9 and human chromosome 11. The mRNA abundance of several MMP genes (MMP1, MMP3, MMP10 and MMP12) increased in human SEN(XRA) and SEN(REP) cells, regardless of O_2_ concentration, and in mouse SEN(XRA) cells in 3% O_2_, relative to human and mouse PRE cells. However, the expression of these genes remained unchanged in SEN(OXI) mouse fibroblasts (Fig. 4D). The expression of MMP3 by SEN(XRA) human [24], [26] and mouse cells was confirmed at the protein level by intracellular immunostaining, and by zymography and western blotting using CM (Fig. 4D).

At least two other gene clusters were coordinately induced upon senescence. The cytokine antibody arrays showed that several members of the CXCL locus (mouse chromosome 5, human chromosome 4) and CCL locus (mouse chromosome 11, human chomosome 17) comprised the SASP (Figs. 4E, S4A; see also Figs. 1A, S1A, S1C). Consistent with other SASP factors, CCL and CXCL family members increased at the levels of mRNA and secreted protein in MEFs that senesced in 3%, but not in 20%, O_2_ (Fig. 4E) and in human SEN(REP) and SEN(XRA) fibroblasts (Fig. S4). These results suggest that the increased expression of both mouse and human SASP genes can involve large chromosomal segments.

### SASPs Stimulate Malignant Epithelial Cell Proliferation

**Figure 5:**
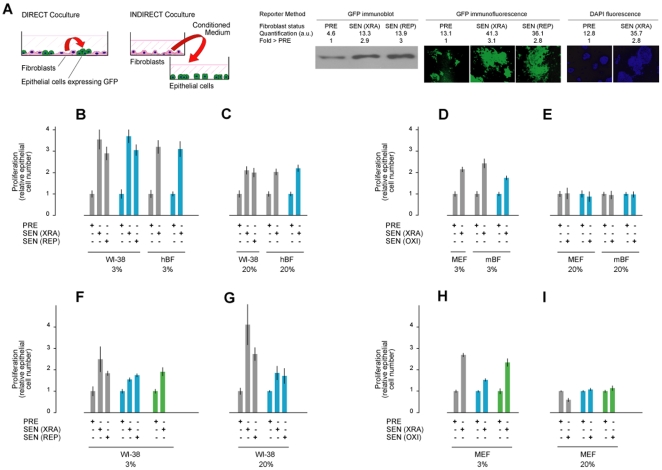
Biological activities of SASPs. A) Diagrams of direct and indirect epithelial/fibroblast co-culture models, and illustration of quantification methods. Most epithelial cells were transfected with GFP. In both co-culture systems, GFP fluorescent of live cells, or western analysis of GFP protein levels, were used to assess epithelial cell growth. Alternatively, in indirect co-culture, epithelial cell proliferation was quantified by fluorescence of DAPI-stained nuclei [25], [54]. B-E) SCp2 (gray) and EpH4v (blue) epithelial cells were co-cultured directly with PRE or SEN cells of human (B–C) or mouse (D–E) origin, and monitored for growth. F–I) SCp2 (gray), EpH4v (blue), and MCF10A (green) epithelial cells were co-cultured with CM from PRE or SEN cells of human (F–G) or mouse (H–I) origin, and monitored for growth.

Human SASPs can disrupt epithelial organization and promote premalignant epithelial cell growth in culture [15], [24], [25], [26], [27]. To determine whether mouse SASPs have these biological activities, we compared the effects of mouse and human fibroblasts on the growth of pre-malignant or malignant mammary epithelial cells (mouse SCp2 and EpH4-v; human MCF10A and ZR75.1) expressing green fluorescent protein (GFP). We assessed epithelial cell proliferation in direct or indirect co-cultures by monitoring GFP expression or fluorescence, or fluorescence from DAPI-stained nuclei (Fig. 5A). In direct co-cultures (Fig. 5B–E), mouse epithelial cells were mixed with human PRE or SEN(XRA or REP) fibroblasts (strains WI-38 or hBF) [15] (Fig. 5B,C), or mouse PRE or SEN(XRA or OXI) fibroblasts (MEFs or mBFs) (Fig. 5D,E). The cultures were maintained at 3% or 20% O_2_, and epithelial proliferation was determined several days later. Compared to PRE cells, SEN human fibroblasts stimulated epithelial cell proliferation regardless of O_2_ concentration (Fig. 5B,C). Mouse fibroblasts behaved similarly, but only when they senesced in 3%, not 20%, O_2_ (Fig. 5D,E). Similar results were obtained using indirect co-cultures in which either mouse or human epithelial cells were cultured with fibroblast CM (Fig. 5F–I). CM from SEN human cells were consistently stimulatory, regardless of the culture O_2_ concentration, whereas CM from mouse cells stimulated epithelial cell proliferation only when the cells were made senescent in 3% O_2_. These findings support the idea that biological activities of the mouse and human SASPs are conserved, and that mouse cells do not express a SASP when the senesce (arrest growth) in 20% O_2_.

### CXCL-1 (GRO-α/KC) Is a Major Conserved Factor That Stimulates Epithelial Cell Growth

**Figure 6:**
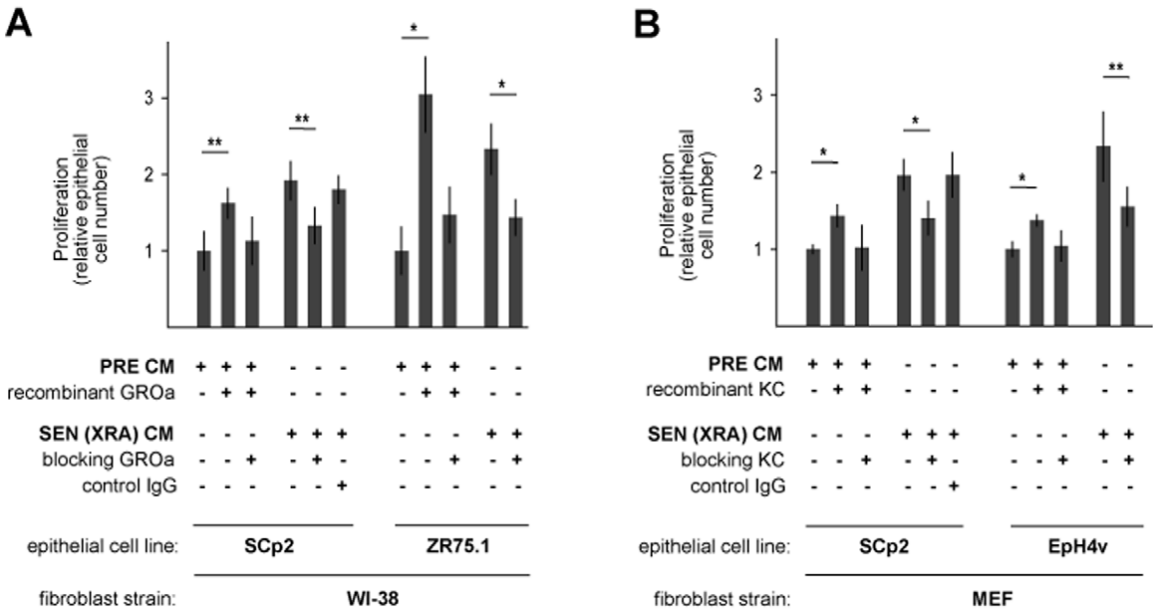
GRO-α/KC is key for the growth promoting effects human and mouse SASPs. A–B) Epithelial cells were incubated with CM from the indicated fibroblasts and cell number was determined by cell counting, total protein content, or GFP fluorescence as described in the legend to Fig. 5. CM from human (A) or mouse (B) cells was used alone or supplemented with GRO-α (A) or KC (B) recombinant protein or with a blocking antibody. Cell growth was significantly stimulated by recombinant protein and inhibited by blocking antibody (*p<0.02; **p<0.05).

To better understand the conserved pro-oncogenic activities of SASPs, we tested specific factors as candidates for stimulating the growth of pre-malignant or malignant epithelial cells. IL-6, IL-8 and CXCL-1 (human GROα) are among the most highly and consistently secreted human SASP factors [15] (Fig. S1C), and overlap with the similarly secreted mouse SASP factors IL-6 and CXCL-1 (mouse KC) (Figs.1A, 2C, S1A). Although neither the IL-6 nor IL-8 in SEN CM stimulated malignant epithelial cell proliferation (Fig. S5A), CXCL-1, a known epithelial cell growth factor [44], was potent in this regard. PRE CM, whether human (Fig. 6A) or mouse (Fig. 6B), supported basal epithelial cell growth; for mouse cells, neither O_2_ nor spontaneous immortalization affected secreted CXCL-1 (KC) levels (Fig. S5B) or growth stimulatory activity (Fig. S5C). However, recombinant GROα or KC, added to PRE CM at concentrations comparable to those in SASPs (determined by ELISA), stimulated epithelial cell growth by 175–300% (Fig. 6A,B). Conversely, addition of GROα or KC blocking antibodies to SEN CM significantly reduced growth stimulatory activity (Fig. 6A,B). These findings identify CXCL-1 as a key conserved SASP factor responsible for stimulating the growth of premalignant and malignant epithelial cells.

### SEN(XRA), but Not SEN(OXI), Mouse Fibroblasts Stimulate Tumorigenesis

**Figure 7:**
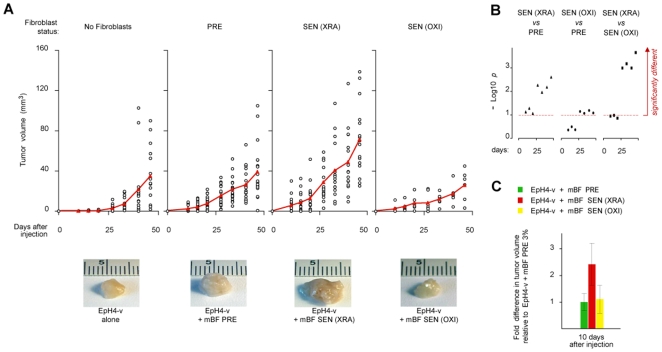
SEN(XRA) but not SEN(OXI) mouse cells promote tumorigenesis *in vivo*. A) EpH4-v epithelial cells were injected into the mammary fat pad area of nu/nu mice alone or with mouse breast fibroblasts made senescent in 3% or 20% O_2_. Tumor volumes were determined as described in Methods. Number of mice used: PRE, 3% O_2_: n = 20; SEN(XRA), 3% O_2_: n = 16; SEN (OXI), 20% O_2_: n = 6; EpH4-v alone: n = 17. B–C) Comparison of average tumor volumes after injection. B) Significant differences between different tumor populations are graphically represented as -Log10(p) where p = 1. Student t-test value, and significance (p>0.9) is shown by the dashed line (see Fig. S6 for all Student t-test values and tumor population comparisons). C) Average fold-differences 10 d after injection.

SEN, but not PRE, human fibroblasts can promote tumorigenic progression of pre-malignant or malignant epithelial cells in mouse xenografts [25], [26]. To determine whether this is true for mouse fibroblasts, we injected weakly tumorigenic EpH4-v mouse mammary epithelial cells, with or without mouse mammary fibroblasts (mBF), into the mammary fat pads of female *nu/nu* mice. PRE mouse cells did not significantly stimulate the growth of EpH4-v derived tumors; this was also true for mBFs that senesced after passage in 20% O_2_ [SEN(OXI)] (Figs. 7A–C, S6A). By contrast, mBFs induced to senesce by XRA in 3% O_2_ [SEN(XRA)] significantly stimulated tumor formation (Fig. 7A–C, S6A). In agreement with earlier findings [45], tumors that formed in the presence of PRE fibroblasts were less vascularized (assessed by von Willebrand Factor (vWF) expression) than those formed in the presence of SEN(XRA) fibroblasts. Notably, however, tumors that formed in the presence of SEN(OXI) fibroblasts were the least vascularized – less so, even, than tumors that formed without fibroblasts (Fig. S6B). This finding is consistent with SEN human and SEN(XRA) mouse cells, but not PRE or SEN(OXI) cells, secreting many factors that can stimulate angiogenesis (Figs. 1A,2C,S1A,S1C).

Together, our results indicate that the SASP is conserved between human and mouse cells, but is abolished by hyperphysiological O_2_, to which mouse cells are much more sensitive.

## Discussion

Mice are used extensively to model many human diseases, including cancer, despite mouse-human differences in fundamental processes such as telomere biology and cellular senescence [37], [46], [47], [48], [49]. Notably, oxygen sensitivity severely limits the growth of mouse, but not human, cells under standard (hyperphysiological O_2_) culture conditions [4]. We show here that this O_2_ hypersensitivity severely affects the secretory profile of mouse cells. Hyperphysiological O_2_ causes murine cells to arrest in a state termed senescence, but the phenotype of such cells differs from that of senescent human cells – and mouse cells that senesce under lower O_2_.

Although mouse cells failed to develop a human-like SASP when cultured in 20% O_2_, the SASPs overlapped substantially when mouse cells were cultured and induced to senesce in physiological O_2_. Under these conditions, the mouse and human SASPs showed substantial qualitative and quantitative conservation. In both cases, prominent secreted factors included inflammatory cytokines (e.g., interleukins) and growth factors and regulators (e.g., IGFBPs, GROα/KC), and shed or soluble forms of adhesion molecules and cell surface receptors (e.g., TNFRs, CAMs). We show further that the human and mouse SASPs include several MMPs. The genes encoding these MMPs form a contiguous gene cluster in both the mouse and human genomes. Of interest, MMPs encoded near the center of these clusters were most highly secreted, whereas those at the 3′ and 5′ ends were expressed at lower levels (Fig. 4D). Two other families of conserved SASP factors (the CXCL and CCL families) are also organized as contiguous gene clusters. These findings suggest that activation of the SASP may entail remodeling of large chromosomal segments, a possibility we are currently exploring.

In addition to conservation at the level of individual factors, biological activities of the SASP were also conserved between mice and humans. Both SASPs stimulated the growth of premalignant and malignant epithelial cells in co-culture models. Moreover, mouse fibroblasts that expressed a SASP stimulated tumor growth in mice, similar to senescent human fibroblasts [25]. We found that much of the SASP growth stimulatory activity was due to secreted CXCL-1 (human GROα, mouse KC). This potent epithelial growth factor has also been shown to induce or reinforce the senescence growth arrest [50], similar to recently reported activities of the SASP factors IL-6 and IL-8 [28], [30].

The fact that some SASP factors help maintain the senescence growth arrest reinforces the apparently paradoxical effects of the senescence response. The senescence growth arrest is an important mechanism for preventing the growth of damaged cells, which are at risk for malignant transformation [3]. In this regard, activity of SASP factors to reinforce the growth arrest is likely beneficial. Further, the SASP may allow damaged cells to communicate their compromised state to surrounding cells [13] in order to stimulate tissue repair [32]. However, when senescent cells are chronically present, which appears to be the case as mammalian organisms age and in several chronic degenerative diseases of aging [17], [18], [19], [20], [21], [22], they may disrupt normal tissue function and drive aging phenotypes and age-related disease, including cancer. This possibility is strengthened by the fact that SASP includes factors that can stimulate inflammation, which underlies many age-related pathologies [23]. Our finding that the mouse and human SASPs share many features, and the identification of culture conditions under which the mouse SASP can be studied, suggests that mice can be used to understand how the beneficial and deleterious effects of the senescence response are balanced.

Our finding that mouse cells that senesce in 20% O_2_ do not develop a SASP adds to the anomalies shown by mouse cells when cultured under hyperoxia. Although under these conditions mouse cells arrest growth and express SA-βGal activity, many of these ‘senescent’ cells continue to synthesize DNA and they retain serum-inducible c-Fos expression. Senescent human cells, and mouse cells made senescent in physiological O_2_, do not share these characteristics. Further, mouse cells that arrest growth in 20% O_2_ do not accumulate persistent DNA damage (53BP1) foci, which mark DNA double strand breaks [51] and correlate with inflammatory cytokine secretion by senescent human cells [13]. Nonetheless, mouse cells that ‘senesce’ in 20% O_2_ accumulate oxidative DNA lesions [4], and such cells are capable of expressing a SASP, albeit weakly, when irradiated (which causes double strand breaks). Others have found that MEFs from 129Sv mice arrest growth in 20% O_2_ and do not spontaneously immortalize but do harbor DNA damage foci [52]. We predict that these cells might also display a SASP, although this was not tested. The mouse SEN(OXI) state displayed by other mouse strains may be a unique senescence growth arrest, dissociated from the senescence secretory program, although it is not clear whether or how this state relates to organismal physiology or pathology.

## Materials and Methods

### Ethics Statement

All procedures used in this study were in compliance with the Public Health Service Policy on Humane Care and Use of Laboratory Animals and incorporated the 1985 U.S. Government Principle. Studies were approved by the Buck Institute's Institutional Animal Care and Use Committee under the protocol #10090. Animals were maintained using the highest possible standard care and priority was given to their welfare above experimental demands at all times.

### Cells

Cells were obtained, cultured and made quiescent or senescent as described [4], [8], [15], [25]. MEFs and mBFs were obtained from C57Bl/6 mice. PRE cultures were defined as showing >75% BrdU or ^3^H-thymidine incorporation over a 24 h labeling interval and <15% SA-βGal staining; SEN cultures, with the exception of SEN(OXI), showed <5% BrdU or ^3^H-thymidine incorporation and >70% SA-βGal staining. To induce SEN(XRA), cells were cultured to confluence, X-irradiated (10 Gy), allowed to recover overnight, then trypsinized and split 1∶3; cells were analyzed 10 d later, when they displayed the enlarged senescent morphology and expressed SA-βgal [8].

### Antibody Arrays

Cultures were washed and incubated in serum-free Dulbecco's modified Eagle medium (DMEM) for 24 h to generate CM. Cells were counted after CM collection. CM were filtered, frozen, and analyzed on antibody arrays (Chemicon; Mouse cat #AA1003M-8) as recommended, with previously described modifications [15].

### ELISA and Immunofluorescence

ELISAs were performed using kits previously described [15]. Immunofluorescence and immunohistochemistry were performed using antibodies and protocols previously described [13], [15], [45].

### Heterotypic Co-Culture and Epithelial Proliferation Assays

Co-culture assays were performed as we previously described [25], [45]. Briefly, epithelial cells (3×10^4^/35 mm dish) were seeded in growth medium containing 1.375 mM CaCl_2_ at least 2 d prior to incubation with CM from PRE or SEN cells, normalized for equal cell number per ml. After 7 d, epithelial cell growth was measured by quantifying GFP or DAPI-stained nuclei, as described [15]. Cellomix high throughput analyses was used to complement measurements in Figs. 5A, 6B and S5A.

### Tumorigenicity Assays

Tumorigenicity assays were performed as previously [25]. Briefly, we injected 5-week-old *nu/nu* mice with a 100 µl suspension of 1×10^6^ EpH4-v cells, with or without 0.75×10^6^ mBFs, subcutaneously under the region of the sixth nipple of the mammary gland. Three fibroblast populations were tested for supporting tumorigenesis: presenescent, cultured in 3% oxygen (PRE; n = 20 mice); senescence induced by irradiation in 3% oxygen (SEN(XRA); n = 16 mice); senescent induced by repeated passages in 20% O_2_ (SEN (OXI); n = 6 mice). EpH4-v cells were also injected alone (n = 17 mice). At the indicated intervals after injection, the three maximum diameters at *x*, *y* and *z* axes were assessed by caliper measurements to determine tumor volume.

### Recombinant Proteins and Blocking Antibodies

Recombinant proteins and blocking antibodies were obtained from RD Systems: human GROα (275-GR; MAB275 and general blocking antibody for GROα/β/γ MAB276), mouse KC (453-KC; MAB4531 and AF-453-NA), and IL-6 (206-IL; MAB2061) and IL-8 (1645 from Sigma; MAB208). A non-specific control antibody from RD Systems was used (Goat IgG AB-108-C).

### Real Time Polymerase Chain Reaction (RT-PCR)

RNA was isolated and analyzed as previously described [15], [45]. cDNA was synthesized using standard methods. Quantitative reverse transcription reactions were done in duplicate or triplicate using SYBR Green PCR master mix (Applied Biosystems, Foster City, CA) and analyzed using an Applied Biosystems 7700 sequence detector. Samples were normalized to the cycle threshold value obtained during exponential amplification of GAPDH, GUS or H1A. Control reactions with RNA or water did not produce significant amplification products.

### Comparative Genomic Hybridization (CGH)

CGH analyses were carried out essentially as previously described [53]. Samples were analyzed using Scanning and OncoBAC arrays. Scanning arrays were comprised of 2464 BACs selected at approximately megabase intervals along the genome.

### Statistical Analyses

Correlation coefficients were evaluated using Pearson's correlation. Statistical significance between distributions of protein signals, tumor size distributions, or growth advantage patterns was evaluated using a Student's T-test with two tails, and an assumption of equal variance. In graphical representation, error bars correspond to standard deviation around the mean. For determination of the significance of overlap, we used the hypergeometric distribution with the following parameters: population size  = 46 (total orthologs proteins on human and mouse array), sample size  = 22 (mouse SASP), successes in population  = 20 (human SASP; see Fig. S1C and our previous report [15]), successes in sample  = 14 (qualitative overlap between the mouse and human SASPs). To determine whether the mouse and human factors are ordered in a similar fashion within each SASP profile, we used the Spearman correlation for ranking order analysis (Dataset S3: H vs M SASP). For this semi-quantitative evaluation, we indistinctively compared all 46 orthologs between mouse or human secretory profiles, assigning the value of their rank to each significantly secreted SASP factor, and a value of 0 to any non-SASP factor (in either human or mouse profiles). Clustering and tree-view analyses were produce using publically available software [43] (http://bonsai.ims.u-tokyo.ac.jp/~mdehoon/software/cluster/software.htm#ctv).

## Supporting Information

Figure S1 SASP of mouse fibroblasts A) Antibody array profile of all mouse cell populations studied, showing an expanded version of Fig. 1A. B) Unsupervised clustering analysis using data presented in Fig. S1A. C) Human SASP profiles, using average values of human embryonic fibroblasts (HEF; WI-38 and IMR90) and human skin fibroblasts (HSF; HCA2 and BJ) induced to senesce by replicative exhaustion or irradiation (see [15]). D) Comparison of secretory profiles of mouse (graph) and human (table) cells made senescent in 3% vs 20% O2. E–F) Comparison between SEN(XRA) SASPs in 3% vs 20% O2 for mouse (E) and human (F) cells.
(3.66 MB TIF)

Figure S2 Comparison between human and mouse orthologs A) Comparison between orthologs found in human cells induced to replicatively senescence in 20% O2 (SEN(REP)) vs mouse cells induced to senesce by replication in 20% O2 (SEN(OXI)). B–C) Comparison using human and mouse orthologues (B), and table of orthologous factors unchanged between PRE and SEN cells (C).
(1.13 MB TIF)

Figure S3 DNA damage in mouse cells and human CGH profiling A) 53BP1 foci in mouse cells irradiated in 20% O2. B) Fraction of 53BP1-positive SEN(OXI) mouse cells that do (BrdU +) or do not (BrdU -) synthesize DNA while growth arrested. C) CGH analysis of human fibroblasts. Pre-senescent and senescent cells (SEN(XRA) or SEN(REP)) do not show significant differences.
(3.18 MB TIF)

Figure S4 mRNA levels from human CXCL and CCL loci A) Human cells, treated as indicated in the legend, were assayed for CXCL and CCL loci mRNA by RT-PCR (complement data to Fig. 4E).
(0.57 MB TIF)

Figure S5 SASP biological activities A) IL-6 and IL-8 are not responsible for promoting epithelial cell proliferation. Epithelial cells were cultured in presence human PRE and SEN CM. Epithelial cells were counted using a Cellomics high throughput reader. Blocking IL-6 or IL-8 antibodies did not reduce cell proliferation. B) Immortal (IM) MEFs do not secrete GROalpha. Shown are antibody array results comparing mouse PRE, SEN and IM cells. C) Immortal (IM) MEFs do not induce proliferation of epithelial cells. The indicated epithelial cells were incubated with the indicated CM and analyzed as described in A.
(1.22 MB TIF)

Figure S6 SASP biological activities during tumorigenesis in vivo A) Table of Student t-test values obtained from comparisons of tumor volumes induced by PRE, SEN(XRA) and SEN(OXI) fibroblasts in mouse xenograft assays. The graph shows the average tumor volumes and standard deviations around the mean. B) Tumor vascularization. Immmunostaining for vWF as a reporter of endothelial cell presence was used to visualize blood vessels. Average vessel numbers per field are reported as small and large vessels; the standard deviation around the average number of all vessels per field is shown.
(2.95 MB TIF)

Dataset S1 Mouse Secretome: Computational analysis of antibody array data presented in Fig. 1 and Fig. S1 (PRE, SEN(XRA), SEN(OXI), and IM mouse fibroblasts cultured in 3% and 20% oxygen condition) The first spreadsheet (“mouse data_raw_ave_fold”) is composed of six main blocks: column A-AT lists the raw antibody array read outs of all 45 mouse cell samples (62 factors); column AW-BK lists the average values for each different group of samples; column BN-CE lists fold average values against the mean within each cell strain; column CH-CY is the log2 of column BN-CE; column DB-EU lists fold of all raw values against the mean of each cell strain; column EX-GQ is the log2 fold of column DB-EU. The second spreadsheet (“mouse data_ttest_log2fold”) lists in column A-AT the data log2 fold of all raw values against the mean of each cell strain (that is column EX-GQ from the first spreadsheet), and calculates in column AV-AY the significance (Student t-test) of variation between subpopulations of interest; finally, the log2 fold average values against the mean of each cell strain (extracted from the column CH-CY in the first spreadsheet) are reorganized as represented in Fig. S1. The third spreadsheet (“Fig. 1A”) extracts only the significant variations from the second spreadsheet (as listed in Fig. 1A). The fourth spreadsheet (“Fig. 1C”) extracts data from the two first spreadsheets and compare directly the SEN(XRA) and SEN(OXI) secretomes. The fifth spreadsheet (“Fig. S1”) rearranges the data as presented in Fig. S1. These data can be used for clustering and correlative analysis.
(0.42 MB XLS)

Dataset S2 Gene Orthology: List of human and mouse genes corresponding to the specific proteins detected by antibody array The first spreadsheet (“human gene IDs + mouse orthologs”) lists all human genes found on the human antibody arrays and their corresponding mouse orthologs. The second spreadsheet (“mouse gene IDs + human orthologs”) lists all mouse genes found on the mouse antibody arrays and their corresponding human orthologs.
(0.09 MB XLS)

Dataset S3 H vs M SASP: Computational analysis of human and mouse SASPs data presented in Fig. 2 and Fig. S2 The first spreadsheet (“human vs mouse SASPs”) lists all human secreted factors and their related t-test values, and lists in parallel all corresponding mouse orthologs and secretory profiles and t-test values. Conversely, the second spreadsheet (“mouse vs human SASPs”) lists all mouse secreted factors and their related t-test values, and lists in parallel all corresponding human orthologs and secretory profiles and t-test values. Only SEN(XRA) at 3% is considered here. In the third spreadsheet (“h&m SASPs ranking (1)”), spreadsheets 1 and 2 are merged to obtain the ranking order of all significantly altered mouse or human SASP factors. The non-SASP factors were given a value of 0; SASP factors were assigned the value of their rank within their population (either human or mouse). The fourth spreadsheet (“h&m SASPs ranking (2)”) shows the direct calculation for Spearman ranking correlation, using the square value of the difference of ranking between human and mouse orthologous secretory profiles.
(0.08 MB XLS)
