# Supplementary figures and images for: A Human-Like Senescence-Associated Secretory Phenotype Is Conserved in Mouse Cells Dependent on Physiological Oxygen
Source: PLoS One. 2010 Feb 12;5(2):e9188. doi: 10.1371/journal.pone.0009188 (PMC2820538; doi:10.1371/journal.pone.0009188)

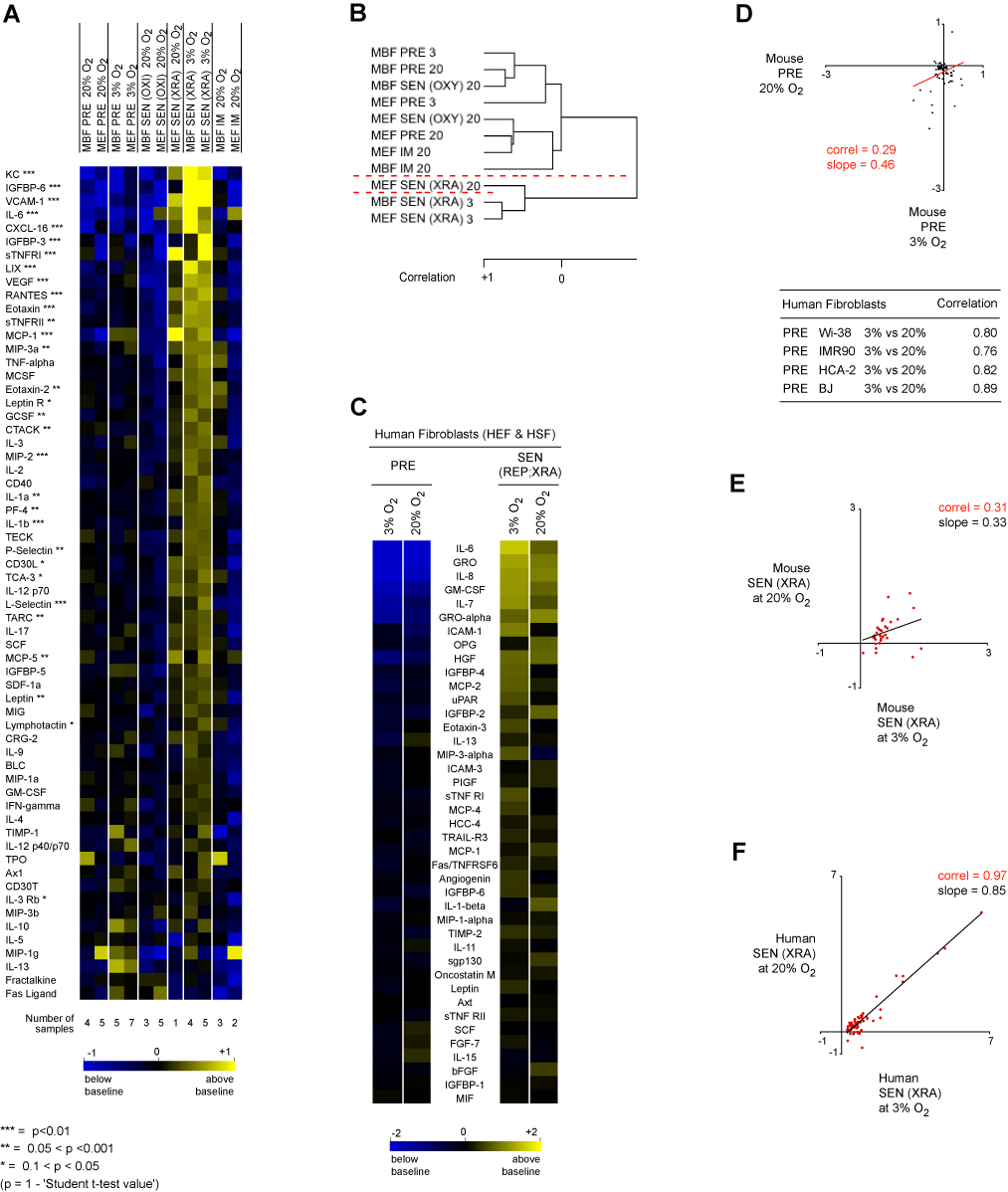

Supplement: Figure S1 — SASP of mouse fibroblasts A) Antibody array profile of all mouse cell populations studied, showing an expanded version of Fig. 1A. B) Unsupervised clustering analysis using data presented in Fig. S1A. C) Human SASP profiles, using average values of human embryonic fibroblasts (HEF; WI-38 and IMR90) and human skin fibroblasts (HSF; HCA2 and BJ) induced to senesce by replicative exhaustion or irradiation (see [15]). D) Comparison of secretory profiles of mouse (graph) and human (table) cells made senescent in 3% vs 20% O2. E–F) Comparison between SEN(XRA) SASPs in 3% vs 20% O2 for mouse (E) and human (F) cells. (3.66 MB TIF) [file pone.0009188.s001.tif]

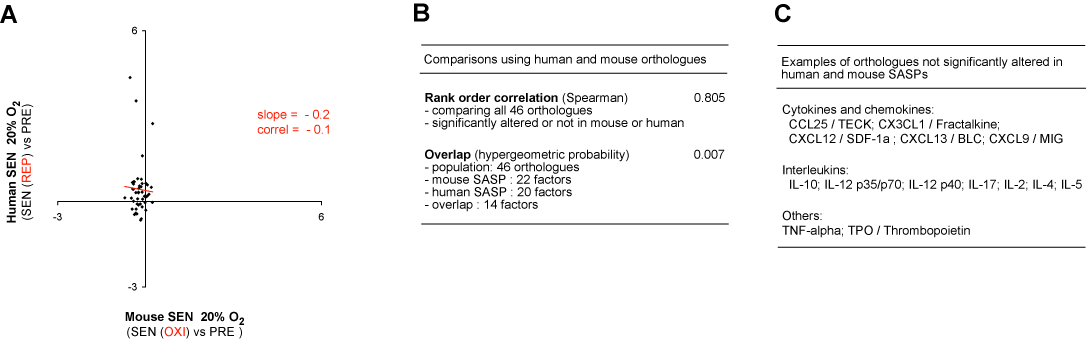

Supplement: Figure S2 — Comparison between human and mouse orthologs A) Comparison between orthologs found in human cells induced to replicatively senescence in 20% O2 (SEN(REP)) vs mouse cells induced to senesce by replication in 20% O2 (SEN(OXI)). B–C) Comparison using human and mouse orthologues (B), and table of orthologous factors unchanged between PRE and SEN cells (C). (1.13 MB TIF) [file pone.0009188.s002.tif]

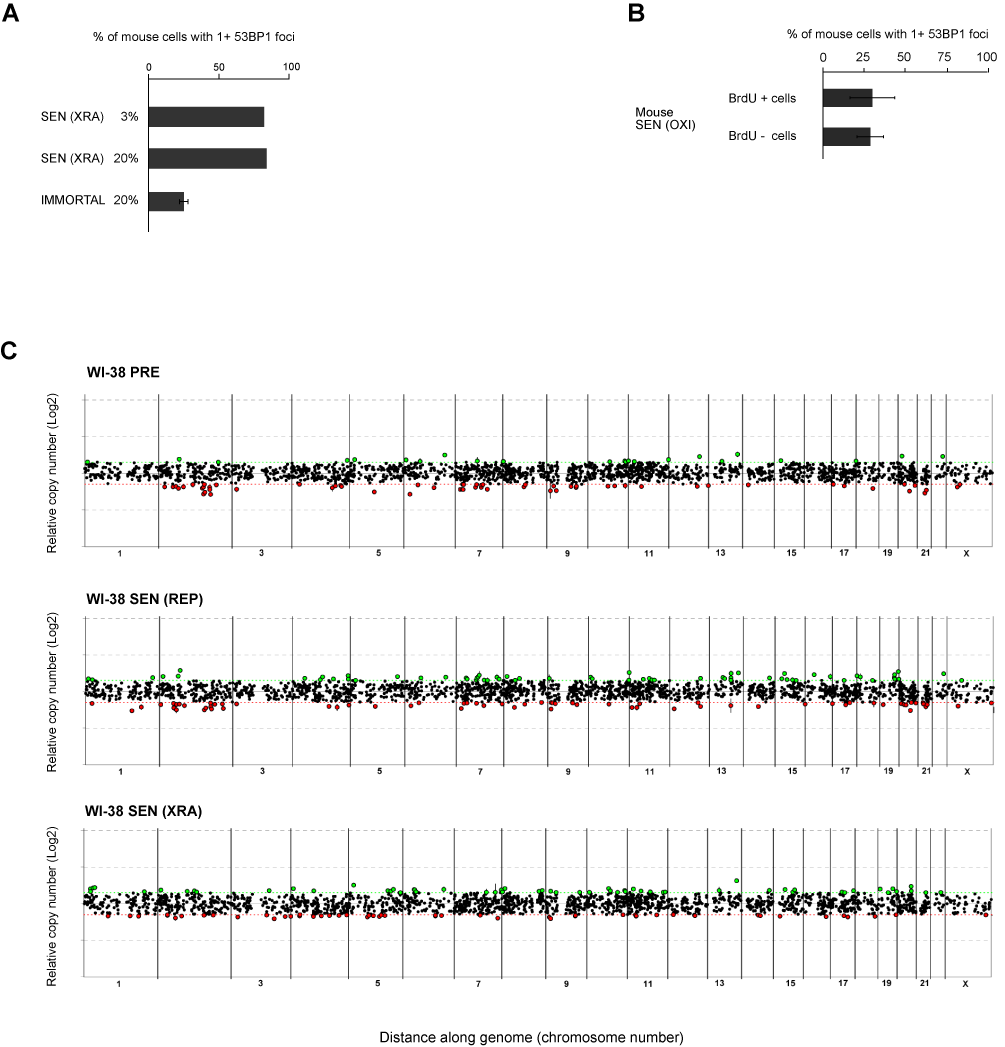

Supplement: Figure S3 — DNA damage in mouse cells and human CGH profiling A) 53BP1 foci in mouse cells irradiated in 20% O2. B) Fraction of 53BP1-positive SEN(OXI) mouse cells that do (BrdU +) or do not (BrdU -) synthesize DNA while growth arrested. C) CGH analysis of human fibroblasts. Pre-senescent and senescent cells (SEN(XRA) or SEN(REP)) do not show significant differences. (3.18 MB TIF) [file pone.0009188.s003.tif]

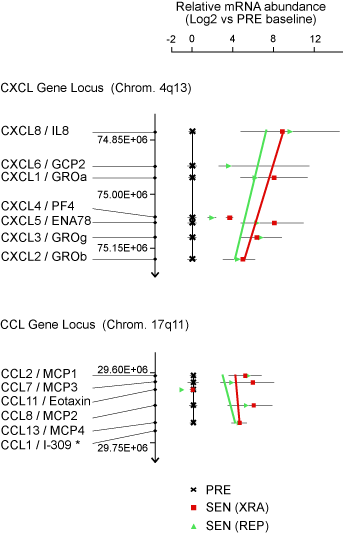

Supplement: Figure S4 — mRNA levels from human CXCL and CCL loci A) Human cells, treated as indicated in the legend, were assayed for CXCL and CCL loci mRNA by RT-PCR (complement data to Fig. 4E). (0.57 MB TIF) [file pone.0009188.s004.tif]

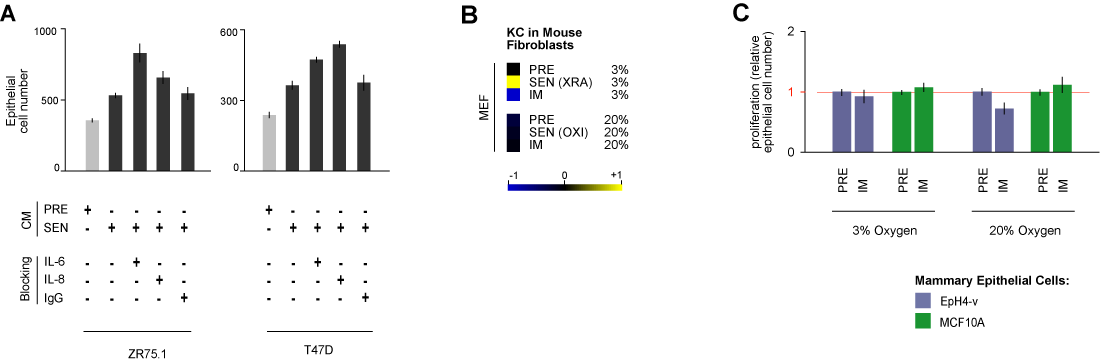

Supplement: Figure S5 — SASP biological activities A) IL-6 and IL-8 are not responsible for promoting epithelial cell proliferation. Epithelial cells were cultured in presence human PRE and SEN CM. Epithelial cells were counted using a Cellomics high throughput reader. Blocking IL-6 or IL-8 antibodies did not reduce cell proliferation. B) Immortal (IM) MEFs do not secrete GROalpha. Shown are antibody array results comparing mouse PRE, SEN and IM cells. C) Immortal (IM) MEFs do not induce proliferation of epithelial cells. The indicated epithelial cells were incubated with the indicated CM and analyzed as described in A. (1.22 MB TIF) [file pone.0009188.s005.tif]

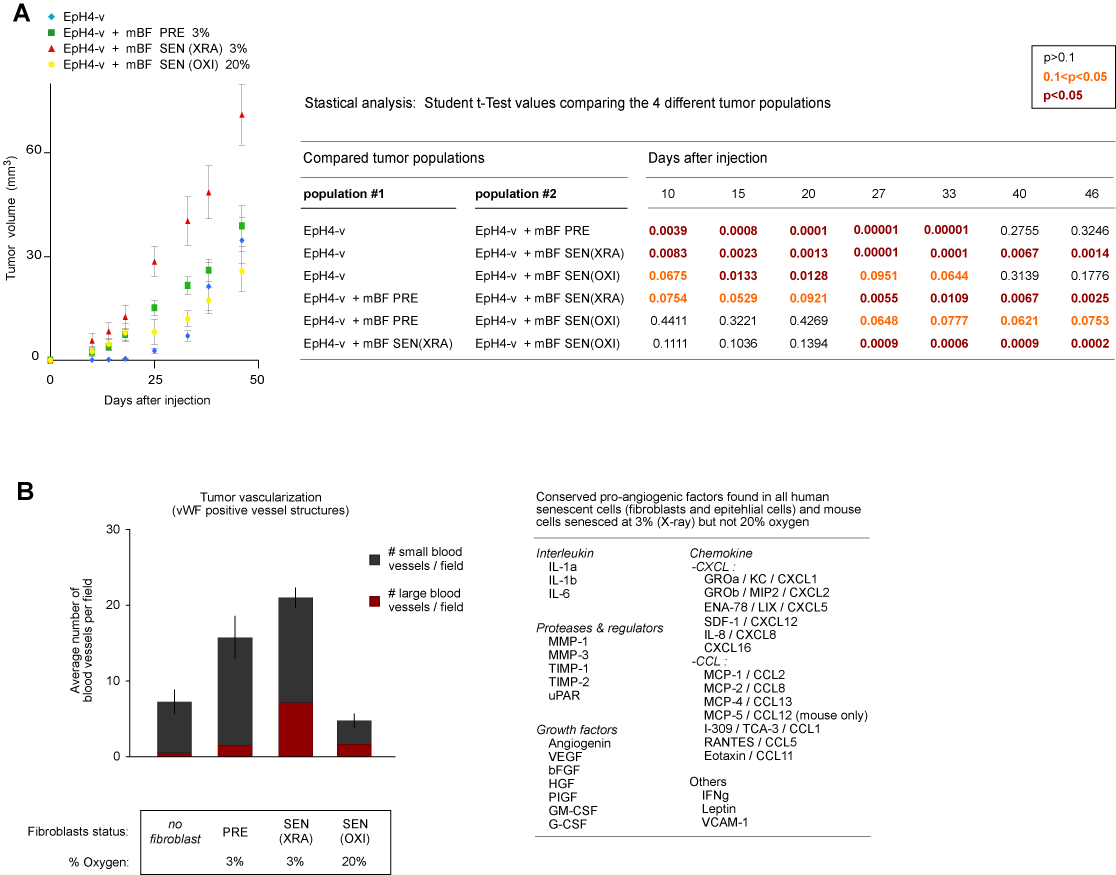

Supplement: Figure S6 — SASP biological activities during tumorigenesis in vivo A) Table of Student t-test values obtained from comparisons of tumor volumes induced by PRE, SEN(XRA) and SEN(OXI) fibroblasts in mouse xenograft assays. The graph shows the average tumor volumes and standard deviations around the mean. B) Tumor vascularization. Immmunostaining for vWF as a reporter of endothelial cell presence was used to visualize blood vessels. Average vessel numbers per field are reported as small and large vessels; the standard deviation around the average number of all vessels per field is shown. (2.95 MB TIF) [file pone.0009188.s006.tif]
